# Assessment of Junior Doctor performance: a validation study

**DOI:** 10.1186/1472-6920-13-129

**Published:** 2013-09-21

**Authors:** Sandra E Carr, Antonio Celenza, Fiona Lake

**Affiliations:** 1 Education Centre, Faculty of Medicine, Dentistry and Health Sciences, University of Western Australia, MB 515 35, Stirling Hwy, Crawley 6009, Western Australia, Australia; 2 School of Primary, Aboriginal and Rural Health, Faculty of Medicine, Dentistry and Health Sciences, University of Western Australia, MB 515 35, Stirling Hwy, Crawley 6009, Western Australia, Australia; 3 School of Medicine and Pharmacology, Faculty of Medicine, Dentistry and Health Sciences, University of Western Australia, MB 515 35, Stirling Hwy, Crawley 6009, Western Australia, Australia

**Keywords:** Educational assessment, Undergraduate medical education, Internship

## Abstract

**Background:**

In recent years, Australia has developed a National Junior Doctor Curriculum Framework that sets out the expected standards and describes areas of performance for junior doctors and through this has allowed a national approach to junior doctor assessment to develop. Given the significance of the judgments made, in terms of patient safety, development of junior doctors, and preventing progression of junior doctors moving to the next stage of training, it is essential to develop and validate assessment tools as rigorously as possible. This paper reports on a validation study of the Junior Doctor Assessment Tool as used for PGY1 doctors to evaluate the psychometric properties of the instrument and to explore the effect of length of experience as a PGY1 on assessment scores.

**Methods:**

This validation study of the Australian developed Junior Doctor Assessment Tool as it was used in three public and other associated hospitals in Western Australia for PGY1 across a two year period addressed two core aims, namely: (1) to evaluate the psychometric properties of the instrument; (2) to explore the effect of length of experience as a PGY1 on assessment scores.

**Results:**

The highest mean scores were for professional behaviours, teamwork and interpersonal skills and the lowest were for procedures. Most junior doctors were assessed three or more times and scores were not different in the first rotation compared to subsequent rotations. While statistically significant, there appeared to be little practical influence on scores obtained by the number of times they were assessed. Principal component analysis identified two principal components of junior doctor performance are being assessed rather than the commonly reported three. A Cronbach Alpha of .883 was calculated for the 10 item scale.

**Conclusions:**

Now that the components of the tool have been analysed it will be more meaningful and potentially more influential to consider these factors on the potential educational impact of this assessment process for monitoring junior doctor development and progression.

## Background

The need to assess junior doctors’ performance in the work place is well recognised. It is important for safe patient care that the small minority of junior doctors, whose performance is giving cause for concern, are identified and addressed. Moreover, formal assessment could ensure all junior doctors receive feedback about their performance in the workplace early in their career, essential for professional development.

A range of approaches are available to assess competence in medical practice [1,2]. Most assessments of competence include direct observation of practice in the clinical setting enabling the assessment of the interrelated domains of competence, namely: medical knowledge, scientific enquiry, clinical skills and patient care, professionalism, communication and interpersonal skills, knowledge of the health system and learning through reflective practice [3]. Multisource feedback is increasingly used in postgraduate medicine to contribute to the assessment of performance [4]. Multi-source feedback is useful in identification of high, intermediate, and low-performing junior doctors and been useful for providing specific formative feedback [5].

It is vital that reliable, valid, feasible and effective measures of performance are used in the assessment and feedback process [6]. Given the significance of the judgments made, in terms of patient safety, development of junior doctors, and preventing progression of junior doctors moving to the next stage of training, it is essential to develop and validate assessment tools as rigorously as possible. Optimising any assessment tool or programme requires consideration of how reliability and validity balance with the other measures of utility such as feasibility and acceptability. Although there is growing evidence to support the reliability and validity of different assessment tools used in postgraduate medical education, including multisource feedback tools [7], it is important individual tools are analysed in local settings to guide where efforts should be made in terms of optimising implementation.

In recent years, Australia has developed a National Junior Doctor Curriculum Framework (ACF) that sets out the expected standards and outlines the learning outcomes required of junior doctors [8]. The ACF is built around three learning areas: Clinical Management, Communication, and Professionalism. These areas are further subdivided into learning topics which have been identified as being critical to both safe prevocational practice and a basis for future training. Through this description of these areas of performance the ACF has allowed a national approach to junior doctor assessment to develop [8]. This National approach is aimed at ensuring consistency in experience received, and the quality of the supervision of Junior Doctors and the feedback they receive on their performance. Junior doctor performance of their clinical management, communication and professional skills is assessed during each clinical rotation in the first postgraduate year (PGY1). The primary supervisor of the junior doctor conducts the assessment which is based on direct observation and hopefully feedback from multiple sources about the junior doctor’s performance over a period of time, usually during an eight to ten week attachment working in a particular clinical area.

The assessment tool has been developed by postgraduate medical councils across Australia and aligns with similar such assessments in the UK [9]. The areas for assessment are related to the Australian Junior Doctor Curriculum Framework, rather than the GMC categories. Being based on the UK form means it is likely to have high face validity. Based on the widespread take up of the assessment it also appears to be feasible and acceptable. While some research has been conducted on the ability of the tool to discriminate poor performance [10], there are few published data evaluating the reliability, validity or educational impact of this Junior Doctor Assessment tool. Neither is there any published evaluation of the principle components making up the assessment tool. Therefore questions exist about the tool’s reliability, and validity to assess performance in the cited components of clinical management, professional and communication skills. These questions need to be answered before the scores obtained from this assessment can be used to accurately assess junior doctor performance and before the tool can be used to explore correlations between undergraduate performance in medical school and workplace performance of Australian junior doctors. This paper reports on a validation study of the Junior Doctor Assessment Tool as used for PGY1 doctors.

## Methods

### Context

The assessment tool was developed by the Postgraduate Medical Council of Western Australia [11] to assess performance in three areas, Clinical Management, Communication and Professionalism, through 10 unique items. The tool has been used at the three tertiary public hospitals, Sir Charles Gairdner Hospital, Fremantle Hospital and Royal Perth Hospital since 2008. This validation study of the Australian developed Junior Doctor Assessment Tool as it was used in three public and other associated hospitals in Western Australia for PGY1 across a two year period addressed two core aims, namely: (1) to evaluate the psychometric properties of the instrument; (2) to explore the effect of length of experience as a PGY1 on assessment scores.

### Study population

Two groups of senior medical students from Years 5 and 6 of the same six year undergraduate curriculum (n = 302) were asked to participate in a longitudinal study following the students until the end of PGY1. The mean age was 23 years at the beginning of the study with 169 (56%) female and 133 (44%) male. Human Research Ethics Committee approval was obtained from the University of Western Australia and the individual public hospitals the graduands would be working in for their first postgraduate year. The study was explained to the group in large face-to-face sessions and individual written consent was obtained.

### Tool description

#### Outcome measure

In Western Australia, the Junior Doctor Assessment Tool is completed by the supervising clinician at the end of each 10 week rotation or attachment. As depicted in Table 1 the junior doctor is assessed using a five-point Likert type scale where 0 = not observed, 1 = below expected level, 2 = borderline, requires assistance, 3 = at expected level and 4 = better than expected. Each item was assigned a score of between 1 and 4 with no value being given to ‘not observed’ for each of the 10 items and summed to give a score out of 40 for each assessment. If a junior doctor is identified as performing at level 1 or 2, the assessor is asked to provide comments to support their rating.

**Table 1:** Replica of a junior doctor assessment form

		**Below expected level**			
	**Not observed**		**Borderline**	**At expected**	**Better than**
		**Requires substantial assistance**	**Requires assistance**	**level**	**expected**
**CLINICAL MANAGEMENT**					
**1.** Clinical Assessment and Patient Management					
**2.** Procedural Skills					
**3.** Emergency Management					
**4.** Adverse event identification and risk minimisation		*Please support these ratings with comments overleaf.*			
**COMMUNICATION**					

**5.** Interpersonal skills with Patients					
**6.** Team work/Interpersonal skills with others in the health care team.					
**7.** Written communication/Record keeping		*Please support these ratings with comments overleaf.*			
**PROFESSIONALISM**					
**8.** Professional Behaviour *(responsive / reflective / ethical)*					
**9.** Scholarly Practice *(learning / critical thinking)*		*Please support these ratings with comments overleaf.*			
**10.** Doctor’s Role in Society *(manager / role model)*					

In addition to the ratings of the three performance areas (clinical management, communication and professionalism) the assessor rates the junior doctors overall performance during the attachment in the form of a global rating using a four point Likert scale where 1 = below expected level, 2 = borderline- requires development, 3 = at expected level, 4 = above expected level.

Assessors are also asked to document the junior doctor’s strengths, areas for improvement including specific information supporting ratings of borderline or below expected performance. Additionally, assessors are asked to comment on whether they have made this assessment based on close personal observation, their general impression of the junior doctor and whether colleagues and other health professional staff have informed the assessment made. This validation study has only included quantitative data pertaining to the three components of Clinical Management, Communication and Professionalism in the analysis.

#### Independent variables

The independent demographic variables included in this study were timing of the assessment (first, second, third, fourth or fifth rotation) and the number of assessments completed over the 12 month period.

#### Data collection

The data of junior doctor performance were collected directly from the medical administration departments in the public hospitals where the junior doctors were employed in the first postgraduate year by the researcher over a two year period and imported to SPSS V20 for statistical analysis procedures.

### Data analysis

Assessments were removed from the analysis if a rating for each of the 10 items was not recorded. This resulted in 822 individual assessments with complete ratings for the 10 items recorded. A qualitative review of assessments suggested assessors applied the ‘not observed’ category for different reasons. Sometimes the assessors checked ‘not observed’ when the junior doctors had not obtained experience in that skill area but in other instances it was used to indicate their lack of engagement in the clinical workplace [12]. In most instances the assessor included a comment to support their use of the ‘not observed’ category. However, no instructions appear to have been given to assessors on when or how to use the “not observed’ category so how to complete the form was left open to interpretation. Therefore, the analysis was conducted excluding ratings of “not observed” such that 134 of the 822 assessments were not considered reliable for inclusion leaving 688 assessments in the analysis.

To address the first objective of this study, that is, to investigate the psychometric properties of the Junior Doctor Assessment instrument, Cronbach’s Alpha reliability coefficient with an item-total scale correlation (to check if any item in the set was inconsistent and therefore could be discarded), and interscale correlation analyses were completed [13]. For item reduction and exploring the factor structure of the instruments, a principal components analysis was conducted with an extraction criterion of Eigenvalue > 1 and with varimax rotation (orthogonal). Items were grouped under the factor where they displayed the highest factor loading. Subsequently, the factor structure was subjected to reliability analysis using Cronbach’s alpha. A Cronbach’s alpha of at least 0.70 was pre-determined to offer an indication of satisfactory internal consistency reliability of each factor. An item-total correlation coefficient of 0.3 or more was considered as adequate evidence of homogeneity and hence reliability [14].

To address the second objective of the study, to quantify the potential influences of the independent variables, descriptive statistics were used to summarise performance for each of the 10 assessment items, plus the overall combined score. This was followed by an ANOVA to test the null hypothesis for the variables of ‘number of assessments’ and ‘length of experience’ (whether it was the first or last rotation in the year).

## Results

### Respondents

Of the 302 medical students, 237 consented to participate in the study (78%). Of these 237, data were available for collection from 200 junior doctors over the two year period (84% of the consented participants). The mean age of participants was 23 years (SD 2.3, range 20–37 years). The total number of assessments completed of the 200 junior doctors included in this analysis was 822 individual assessments. The proportion of females in the respondent group was 54%, representative of the population of graduands. There was no significant difference identified in descriptive scores for the two cohorts of respondents for the 10 items, therefore the findings of both cohorts are reported together.

### Descriptive findings and effect of timing of rotation

As illustrated in Table 2 the lowest mean scores were obtained for the items pertaining to the ability to perform procedures, Emergency Management and the Doctor’s role in Society. The highest mean scores were observed for the items pertaining to abilities around professional behaviour, interpersonal skills, teamwork and written communication skills.

**Table 2:** Descriptive statistics of each item

**Items ranked in order of highest to lowest mean score**	**Mean (SD)**
Teamwork in the health care team	3.67 (0.48)
Interpersonal skills with patients	3.60 (0.49)
Professional behaviour	3.58 (0.50)
Clinical assessment and patient management	3.50 (0.51)
Written communication	3.48 (0.50)
Scholarly practice	3.35 (0.48)
Adverse event identification	3.34 (0.49)
Doctor’s role in society	3.31 (0.47)
Emergency management	3.27 (0.45)
Procedural skills	3.21 (0.42)

Most of the junior doctors were assessed three or more times (92%) in the first postgraduate year with only 1% assessed once and 7% assessed twice. As illustrated in Table 3, there was a small significant difference in the overall mean score obtained with the increasing number of times they were assessed (F = 2.020, p = 0.014). However, there were no observed effects of the amount of experience obtained (F = 1.170, p = 0.294). That is, the observed overall mean score obtained was not significantly different in the first rotation of the year compared with any of the other subsequent rotations of the year.

**Table 3:** Influence of number of assessments and rotation (experience) on overall combined score

	**Category (count)**	**Overall combined score**	**F (significance)**
		**Mean (SD)/40**	
*Number of assessments (n = 688)*	Once (8)	33.9 (3.4)	2.020 (.014*)
	Twice (48)	34.0 (3.2)	
	Three times (136)	34.1 (3.3)	
	Four times (194)	34.3 (3.2)	
	Five times (302)	34.6 (3.4)	
*Rotation (n = 688)*	First (160)	34.2 (3.3)	1.170 (.294)
	Second (167)	34.2 (3.4)	
	Third (146)	34.8 (3.3)	
	Fourth (130)	34.5 (3.4)	
	Fifth (102)	34.5 (3.2)	

*p <0.05.

### Dimension structure and reliability of the dimensions

A Cronbach Alpha of 0.883 was obtained for the 10 item scale with an Alpha of 0.786 for the items within the pre-existing Communication subscale, 0.776 for the Clinical Skills subscale and 0.759 for the subscale for Professionalism.

The 10 items were subjected to a principal components analysis (PCA) using SPSS Version 20 as summarised in Table 4. Prior to performing PCA, the suitability of the data for factor analysis was assessed. Inspection of the correlation matrix revealed the presence of many coefficients of 0.3 and above. The Kaiser-Meyer-Oklin value was 0.912, exceeding the recommended value of 0.6 [14] and Bartletts Test of Sphericity reached significance, supporting the factorability of the correlation matrix [14]. Principal component analysis yielded 2 factors with an Eigen value greater than 1, in total explaining 49.7% and 10.7% of the variance respectively. An inspection of the screeplot revealed a clear break after the second component. The factors (all with factor loadings of > 0.4) comprised six items that have been labelled “Clinical Management subscale” which explained 49.7% of the variance and four items labelled “Communications subscale” that explained 10.7% of the variance. There was a positive correlation between the two factors (r = 0.702).

**Table 4:** Pattern and structure matrix for PCA with Varimax rotation of junior doctor assessment tool items

**Item**	**Rotated**		**Communalities**
	**Component 1**	**Component 2**	
Emergency management	**0.795**		0.659
Adverse event identification	**0.709**	0.326	0.609
Procedural skills	**0.695**		0.498
Doctor’s role in society	**0.666**	0.331	0.553
Scholarly practice	**0.638**	0.366	0.541
Clinical assessment and patient management	**0.529**	0.492	0.521
Teamwork in the health care team		**0.821**	0.712
Interpersonal skills with patients		**0.819**	0.707
Professional behaviour	0.303	**0.760**	0.670
Written communication	0.366	**0.662**	0.573
Cronbach Alpha for components	0.829	0.834	

Note: major loadings for each item are bolded.

Cronbach Alphas for the 10 item scale was 0.883 and was 0.829 for the 6 item ‘Clinical Management subscale’ and 0.834 for the 4 item ‘Communication subscale’, indicating good internal consistency and reliability of the questionnaire in its entirety and for both subscales.

## Discussion

This paper reports a validation study of the Junior Doctor Assessment Tool as used for PGY1 doctors in Australia, as used on Western Australia. Interestingly, the lowest mean scores were obtained for the items pertaining to the ability to perform procedures and emergency management and the highest mean scores were around professional behaviours, interpersonal skills, teamwork and written communication skills. While these findings may reflect the type of clinical work in each attachment and the relationship or type of interaction between the supervisor and junior doctor, they do fit with those reported in the literature on the areas where graduates feel most and least prepared when commencing the first post graduate year [15,16].

While there was a small statistically significant increase in the overall mean score obtained with an increasing number of assessments, this increase is unlikely to be practically applicable. Moreover, the observed overall mean score obtained was not significantly different in the first rotation of the year compared with any of the other subsequent rotations of the year. Therefore it appears that each piece of assessment is independent for that junior doctor in that particular rotation. It is possible the assessors adjust the standard by which they are assessing the junior doctor according to how much experience they have obtained (starting off or nearing the end of their first year), in particular as the descriptor for the performance level where “Most doctors will be in this category” (Category 3), is “At expected level”. It is also possible that the tool is just not sensitive enough to pick up these changes. However, if performance is being assessed in this way then it limits the ability of the assessment process to monitor for the expected development and progression of junior doctor performance over the whole year.

A high level of internal consistency was identified for the items in the assessment tool. This internal consistency is highest when considering the scale in its entirety (0.883), but the Clinical Management subscale with 6 items and the 4 item Communication subscale both have Cronbach Alphas above 0.82.

Together the findings discussed here, point to a tension between the reliability and validity of the tool and echoes similar findings from elsewhere [9,17]. The analysis demonstrated two principal components – rather than the three factors commonly reported, which reflect the way the assessment form is structured, with the questions falling into the three areas of clinical management, communication skills and professionalism. Robust factor structures with good internal consistency were found for two subscales that have been labelled *“Clinical Management”* and *“Communication”.* It would appear that the *Clinical Management* subscale is assessing a combination of knowledge and skills in the area of clinical management while the *Communication* subscale is measuring interpersonal and written communication skills alongside some aspects of professional behaviour. However, exactly how assessors are interpreting some items remains unclear, therefore it may be more valid and reliable to use the 10 items of the scale or individually to comment on the junior doctors’ performance in a particular area. Assessors need to consider which of the 10 items are most important for monitoring development of the junior doctor and whether this differs for different clinical attachments. One recent study suggested that underperformance of junior doctors was more likely to be detected in emergency medicine rotations [18].

Limitations to this study include the loss to follow up of 30 of the original 237 study participants, incomplete data and the difficulties of interpreting the category of ‘not observed’ and of summarising the junior doctor assessment data by combining the scores for the individual assessment items. Despite these limitations, the findings of this study do seem to be validated by the literature. It would increase the generalizability of these findings if other states in Australia replicated this work so as to confirm the component analysis of the tool that has been widely adopted for use.

It is understood factors such as training of assessors, whether assessors ensure multi-source feedback is used, the qualitative aspects of the feedback given to the junior doctors or the gender of the learner can have an effect on the assessment scores obtained or the sensitivity of the assessment to identify the junior doctor with performance difficulties. All these factors need to be optimised so underperforming or incompetent trainees are identified accurately.

## Conclusions

The important finding in this work has shown taking a commonly used tool (WBA from UK and many other countries) and contextualising it to an Australian setting by covering the main components of the Junior Doctor Curriculum Framework, resulted in distorting the way these measures are used to determine learner competence. The components of the tool have been analysed and only two rather than three areas are being used. It will be more meaningful and potentially more influential to consider these factors and the potential resultant educational impact of this assessment process for monitoring junior doctor development and progression through a qualitative analysis. From there we will be able to answer how we utilise this junior doctor performance scores to evaluate relationships between academic and work place based performance.

## Competing interests

The authors declare that they have no competing interests.

## Authors’ contributions

This work was completed by three authors: SEC, AC and FL. SEC, AC and FL were responsible for the study conception and design. SEC performed the data collection and data analysis. SEC was responsible for the drafting of the manuscript. AC and FL made critical revisions to the paper. All authors read and approved the final manuscript.

## Pre-publication history

The pre-publication history for this paper can be accessed here:

http://www.biomedcentral.com/1472-6920/13/129/prepub

## References

[B1] Epstein RM Assessment in medical education N Engl J Med 2007 13 4 387 396 10.1056/NEJMra054784 17251535

[B2] MiIler A Archer J Impact of workplace based assessment on doctors’ education and performance: a systematic review BMJ 2010 doi:10.1136/bmj.c5064*:* 1-6. [Internet].18th February 2013. 10.1136/bmj.c5064 PMC2945627 20870696

[B3] Batalden P Leach D Swing S Dreyfus H Dreyfus S General competencies and accreditation in graduate medical education: an antidote to overspecification in the education of medical specialists Health Aff 2002 13 5 103 111 10.1377/hlthaff.21.5.103 12224871

[B4] Norcini J Burch V Workplace-based assessment as an educational tool: AMEE Guide No. 31 Med Teach 2007 13 855 871 10.1080/01421590701775453 18158655

[B5] Warm EJ Schauer D Revis B Boex JR Multisource feedback in the ambulatory setting J Grad Med Educ 2010 13 269 277 10.4300/JGME-D-09-00102.1 21975632 PMC2941386

[B6] van der Vleuten C Schuwirth L Assessing professional competence: from methods to programmes Med Educ 2005 13 309 317 10.1111/j.1365-2929.2005.02094.x 15733167

[B7] Overeem K Wollersheim HC Arah OA Cruijsberg JK Grol RPTM Lombarts K Evaluation of physicians’ professional performance: an iterative development and validation study of multisource feedback instruments BMC Health Serv Res 2012 13 80 2 11 22448816 10.1186/1472-6963-12-80 PMC3349515

[B8] cpmec Guidelines for Junior Doctors using the National Assessment tools Australia: Confederation of Postgraduate Medical Education Councils[cited 2013 February 17th]. Available from: http://www.cpmec.org.au/index.cfm?Do=View.Page&PageID=198#guidelines

[B9] PMETB Developing and maintaining an assessment system - a PMETB guide to good practice 2007 United Kingdom

[B10] Bingham C Crampton R A review of prevocational medical trainee assessment in New South Wales MJA 2011 13 410 412 21978350 10.5694/mja11.10109

[B11] pmcwa Postgraduate Medical Council of Western Australia: Junior Doctor Assessment Form 2012 [cited 2012 17th October]. Available from: http://www.pmcwa.health.wa.gov.au/home/

[B12] Duvivier RJ van Dalen J Muijtjens A Moulaert V van der Vleuten C Scherpbier A The role of deliberate practice in the acquisition of clinical skills BMC Med Educ 2011 13 Available from: http://www.biomedcentral.com/1472-6920/11/101 10.1186/1472-6920-11-101 PMC3293754 22141427

[B13] Streiner D Norman G Health measurement scales: a practical guide to their development and use. 4th Edition ed 2008 Oxford: Oxford University Press

[B14] Tabachnick BG Fidell L Using multivariate statistics 2007 5 Boston: Pearson Education Inc

[B15] Watmough S O’Sullivan H Taylor D Graduates from a traditional medical curriculum evaluate the effectiveness of their medical curriculum through interviews BMC Med Educ 2009 13 Available from: http://www.biomedcentral.com/1472-6920/9/64 10.1186/1472-6920-9-64 PMC2773762 19857252

[B16] Tallentire VR Smith SE Wylde K Cameron HS Are medical graduates ready to face the challenges of foundation training? Postgrad Med J 2011 13 590 595 10.1136/pgmj.2010.115659 21690255

[B17] Pella G Homer M Robert TS Assessor training: its effects on criterion‒based assessment in a medical context Int J Res Method Educ 2008 13 2 143 154 10.1080/17437270802124525

[B18] Aram N Brazil V Davin L Greenslade J Intern underperformance is detected more frequently in emergency medicine rotations Emerg Med Australas 2013 13 68 74 10.1111/1742-6723.12031 23379455

